# Geographic differences in pharmacotherapy patterns and outcomes of acute ischemic stroke in China

**DOI:** 10.1186/s12883-024-03564-9

**Published:** 2024-02-15

**Authors:** Ying Li, Qianhua Ou, Yuxuan Lu, Zhiyuan Shen, Jieyu Li, Zhuangzhuang Zhang, Liwen Tai, Guozhong Li, Huisheng Chen, Guiru Zhang, Lei Zhang, Xuwen Sun, Jinhua Qiu, Yan Wei, Sainan Zhu, Zhaoxia Wang, Weiping Sun, Yining Huang

**Affiliations:** 1 Department of Neurology, Peking University First Hospital, 8 Xishiku Street, Xicheng District, Beijing, 100034 China; 2 Beijing Key Laboratory of Neurovascular disease discovery, Beijing, China; 3 Department of medical affairs, Techpool Bio-Pharma Co., LTD, Guangzhou, China; 4 Department of Neurology, Second Hospital of Hebei Medical University, Shijiazhuang, China; 5 Department of Neurology, First Affiliated Hospital of Harbin Medical University, Harbin, China; 6 Department of Neurology, General Hospital of Shenyang Military Command, Shenyang, China; 7 Department of Neurology, Penglai People’s Hospital, Penglai, China; 8 Department of Neurology, Fifth Affiliated Hospital of Sun Yat-sen University, Zhuhai, China; 9 Department of Neurology, Qindao University Medical College Affiliated Yantai Yuhuangding Hospital, Yantai, China; 10 Department of Neurology, Huizhou First Hospital, Huizhou, China; 11 Department of Neurology, Harrison International Peace Hospital, Hengshui, China; 12 Department of Biostatistics, Peking University First Hospital, Beijing, China

**Keywords:** Acute ischemic stroke, Geographic variation, Outcome, Pharmacotherapy

## Abstract

**Background:**

Vast economic and healthcare status discrepancies exist among regions in China, contributing to different treatment patterns. This study was aimed to investigate the current status of pharmacotherapy for acute ischemic stroke (AIS) and outcomes in China and explore the geographic variation in stroke care.

**Methods:**

This study was a multicenter prospective registry study, which collected the data of patients with AIS from 80 hospitals in 46 cities in 2015–2017 across China. Poor functional outcome defined as a modified Rankin Scale score of 3–6 was assessed at 3 and 12 months. Multivariate logistic regression was used.

**Results:**

Among 9973 eligible patients, the number of receiving intravenous thrombolysis (IVT), antiplatelet agents, anticoagulants, statin and human urinary kallidinogenase was 429 (4.3%), 9363 (93.9%), 1063 (10.7%), 6828 (74.7%) and 5112 (51.2%), respectively. Multivariable analysis showed IVT use in northeastern was significantly more frequent than in eastern region (OR = 3.17, 95% CI, 2.53–3.99), while the antiplatelets agents use were less frequent (OR = 0.46, 95%CI: 0.38–0.57). The proportions of poor outcomes at 3 and 12 months were 20.7% and 15.8%, respectively. Multivariate analysis showed AIS patients from northeastern and central region had significantly lower risk of poor outcome at month 3 and 12 than those from eastern region (all *P <* 0.05).

**Conclusions:**

There was a low IVT use and a high antiplatelet agent and statin use for AIS in China. The pharmacotherapy and prognosis of AIS had variation by geographic region.

**Trial registration:**

This study was registered with ClinicalTrials.gov (NCT02470624).

## Background

Stroke is the second leading cause of death and a major cause of disability worldwide [1, 2]. In China, acute ischemic stroke (AIS) represents approximately 70% of all strokes, creating a heavy burden on society and the healthcare system, with 1.57 million deaths in 2018 [3].

Although the role of endovascular therapy in acute ischemic stroke (AIS) has been well established, the pharmacotherapy is still an important component of the comprehensive management of acute ischemic stroke. Some medications, for example, aspirin and recombinant tissue plasminogen activator (rt-PA), have been confirmed to improve the prognosis of acute ischemic stroke patients by many studies and recommended in a series of stroke guidelines.

However, there are discrepancies in applying the standard treatment regimens for AIS between China and developed countries [4]. Indeed, about 2.5% of the patients with AIS received intravenous thrombolysis (IVT) in China in 2013 [5–7], compared with 8.1% in the United States of America (USA) [4]. A gap also exists between China and the USA regarding anticoagulation for stroke patients with atrial fibrillation (AF) (21.0% vs. 94.4%) and lipid-lowering treatment (66.3% vs. 95.8%), reported by previous literatures [4, 8, 9]. Recently, the professional community has made many efforts to improve the care of stroke patients in China [8], including releasing Chinese guidelines for AIS and promoting continuing medical education of healthcare providers [10]. Still, beyond the differences between China and other countries, vast economic and healthcare status discrepancies exist among regions in China [11, 12].

This study aimed to describe the current status of pharmacotherapy and prognosis of AIS in China, and explore the geographic differences on the choice of therapeutic drugs for AIS and prognosis of the patients with AIS. This study was based on the Chinese Acute Ischemic Stroke Treatment Outcome Registry (CASTOR) database.

## Methods

### Study design

This study was based on CASTOR database, a multicenter, hospital-based, prospective registry database, collecting data from 80 hospitals in 46 cities across China between 2015 and 2017. The design and protocol of CASTOR study had been described previously [13]. Hospitals included in the study were at least secondary level, mostly tertiary level, which were required to have a neurology ward with over 100 stroke patients admitted each year.

The inclusion criteria were (1) age ≥ 18 years; (2) acute ischemic stroke diagnosed according to the Chinese Guidelines for Diagnosis and Treatment of Acute Ischemic Stroke (2014) [10], (3) admitted within 1 week after stroke onset, and (4) consented to participate in this study. The exclusion criteria were (1) patients with confirmed cerebral hemorrhage, (2) patients with an expected survival less than 3 months due to systemic diseases, or (3) patients can not provide continuous follow-up information.

### Standard protocol approvals, registrations, and patient consents

This study was registered with ClinicalTrials.gov (NCT02470624) and approved by the ethics committees of Peking University First Hospital (IRB approval number: 2015 [922]) and all participating hospitals. Written informed consent was obtained from all patients. Good Clinical Practice guidelines in accordance with the Declaration of Helsinki were followed, and patients’ privacy was strictly protected.

### Data collection and follow-up

The patients underwent five assessments during the study: baseline (at admission), 7 ± 2 days after enrollment, discharge, and 3 and 12 months after onset. The baseline characteristics included demographics (age, sex, and medical insurance) and medical history (hypertension, diabetes, dyslipidemia, AF, history of stroke including previous ischemic stroke and/or hemorrhagic stroke, coronary artery disease, and history of tumors). Clinical features of the index stroke including the National Institutes of Health Stroke Scale (NIHSS) [14] score and the Glasgow Coma Scale (GCS) [15] score were evaluated at baseline. All assessors had received standardized training to ensure the accuracy of the assessment. Stroke etiology was based on the Trial of Org 10,172 in Acute Stroke Treatment (TOAST) classification [16].

**Fig. 1:**
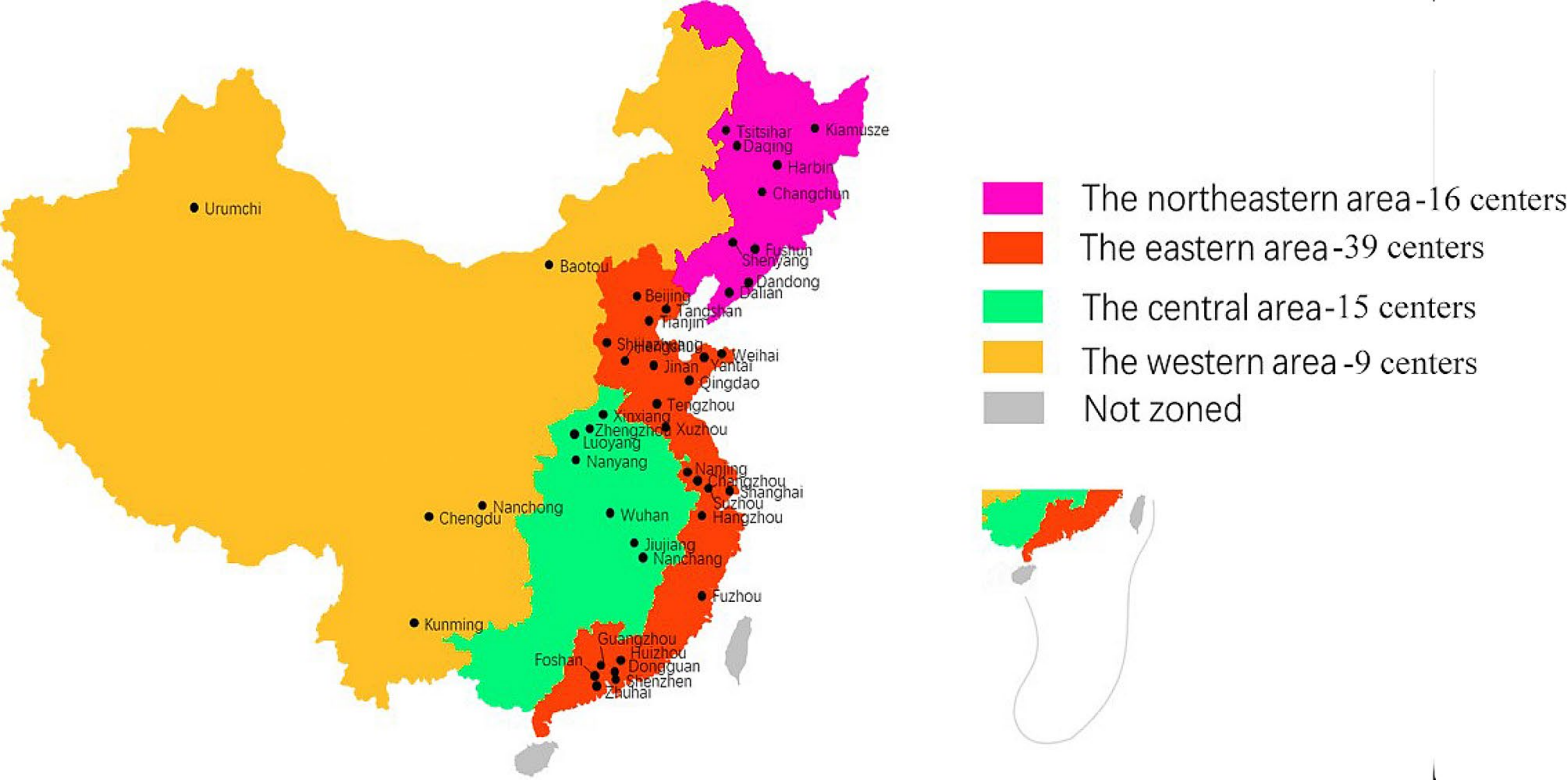
Distribution of participating hospitals

The region was classified as eastern (Beijing, Tianjin, Hebei, Shandong, Jiangsu, Shanghai, Zhejiang, Fujian, and Guangdong), central (Anhui, Henan, Hubei, Hunan, and Jiangxi), western (Xinjiang, Inner Mongolia, Qinghai, Shanxi, Chongqing, Sichuan, Yunnan, and Guangxi), and northeastern (Heilongjiang, Jilin, and Liaoning) region [17] (Fig. 1). In China, hospital level represents the medical level of the hospital, with tertiary level being the best, followed by secondary level and primary level.

The use of medications for AIS during hospitalization were collected from the medical records. IVT agents included rt-PA and urokinase. Antiplatelet agents included aspirin, clopidogrel, tirofiban, cilostazol, and ticagrelor. Anticoagulation agents included heparin, low molecular weight heparin (LMWH), fondaparinux sodium, warfarin, and non-vitamin K oral anticoagulants (NOACs).

Functional outcome was measured with the modified Rankin Scale (mRS) [18] at 3 and 12 months after onset. Poor outcome was defined as a mRS score of 3–6.

### Statistical analysis

Continuous variables were presented as medians (interquartile ranges (IQR)) and analyzed using the Kruskal-Wallis tests. Categorical variables were presented as n (%) and analyzed using the chi-square or Fisher’s exact tests. To estimate the differences among regions in the use of medication for AIS and risk of poor outcomes at 3 and 12 months, univariable and multivariable logistic regression models were used and east region was used as reference. In the multivariable models (enter method), the confounders included patient characteristics such as demographics (age, sex, and medical insurance), medical history (hypertension, diabetes, dyslipidemia, AF, previous stroke, coronary artery disease, and history of tumors), clinical features of the index stroke (the NIHSS score and the GCS score), and hospital level. For all statistical analyses, significance was accepted as *P* < 0.05. All statistical analyses were conducted using SPSS version 23.0 (IBM, Armonk, NY, USA).

## Results

### Baseline characteristics

**Fig. 2:**
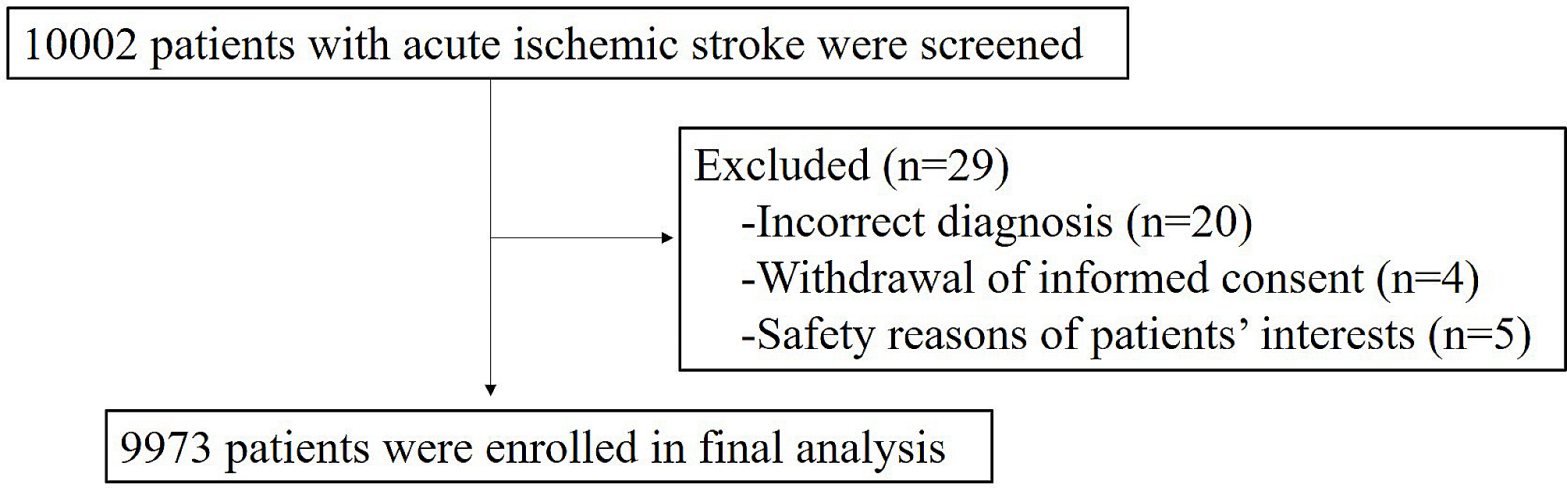
Patient flowchart

A total of 10,002 patients were enrolled in the CASTOR study from 80 hospitals in 46 cities across China. After excluding 29 patients because of incorrect diagnosis (*n* = 20), withdrawal of informed consent (*n* = 4), and safety reasons or patients’ interests (*n* = 5), 9973 patients were included (Fig. 2).

The median age was 64 years, and 34.2% of the patients were female. There were 8218 (82.5%) patients who had medical insurance. There were 4806 (48.2%) patients from the eastern region, 2552 (25.6%) patients from the northeastern region, 2024 (20.3%) patients from the central region, and 591 (5.9%) patients from the western region. There were significant differences in the distributions of age, medical insurance, hypertension, diabetes, dyslipidemia, AF, history of stroke, coronary heart disease, cancer, GCS at baseline, NIHSS at baseline, stroke etiology, and hospital level among the four regions (all *P* < 0.05) (Table 1).

**Table 1:** Baseline characteristics of the patients and hospitals

	All (*N* = 9973)	East (*N* = 4806)	Northeast (*N* = 2552)	Central (*N* = 2024)	West (*N* = 591)	*P*
Age, years, median (IQR)	64 (56–73)	65 (56–73)	63 (56–71)	64 (55–73)	64 (55–74)	0.009
Female, n (%)	3415 (34.2)	1616 (33.6)	861 (33.7)	720 (35.6)	218 (36.9)	0.21
Medical insurance ^a^, n (%)	8218 (82.5)	3805 (79.3)	2129 (83.5)	1837 (90.8)	447 (75.6)	< 0.001
Medical history, n (%)						
Hypertension	6423 (64.4)	3146 (65.5)	1637 (64.1)	1302 (64.3)	338 (57.2)	0.001
Diabetes	2568 (25.7)	1267 (26.4)	683 (26.8)	479 (23.7)	139 (23.5)	0.038
Dyslipidemia	317 (3.2)	190 (4)	33 (1.3)	77 (3.8)	17 (2.9)	< 0.001
Atrial fibrillation	429 (4.3)	204 (4.2)	143 (5.6)	61 (3.0)	21 (3.6)	< 0.001
Previous stroke	2364 (23.7)	929 (19.3)	815 (31.9)	506 (25.0)	114 (19.3)	< 0.001
Coronary heart disease	1373 (13.8)	596 (12.4)	428 (16.8)	284 (14.0)	65 (11.0)	< 0.001
Tumor	243 (2.4)	128 (2.7)	65 (2.5)	45 (2.2)	5 (0.8)	0.049
GCS score ^a^						
Median (IQR)	15 (15–15)	15 (15–15)	15 (15–15)	15 (14–15)	15 (15–15)	< 0.001
13–15, n (%)	8993 (90.2)	4355 (90.6)	2334 (91.4)	1788 (88.4)	516 (87.3)	< 0.001
9–12, n (%)	758 (7.6)	339 (7.1)	170 (6.7)	185 (9.1)	64 (10.8)	
3–8, n (%)	220 (2.2)	110 (2.3)	48 (1.9)	51 (2.5)	11 (1.9)	
NIHSS score ^a^						
Median (IQR)	4 (2–7)	4 (2–8)	4 (2–7)	4 (2–8)	4 (2–8)	< 0.001
≤ 3, n (%)	4489 (45.0)	2110 (43.9)	1273 (49.9)	847 (41.9)	259 (43.8)	< 0.001
4–25, n (%)	5408 (54.2)	2652 (55.2)	1269 (49.7)	1157 (57.2)	330 (55.8)	
≥ 26, n (%)	73 (0.7)	42 (0.9)	10 (0.4)	19 (0.9)	2 (0.3)	
Stroke etiology (TOAST criteria) ^b^						< 0.001
Large artery atherosclerosis, n (%)	2889(64.6)	848(59.0)	832(75.7)	1129(63.2)	80(52.6)	
Cardioembolism, n (%)	191(4.3)	67(4.7)	58(5.3)	55(3.1)	11(7.2)	
Small vessel occlusion, n (%)	1130(25.3)	457(31.8)	101(9.2)	525(29.4)	47(30.9)	
Other, n (%)	105(2.3)	27(1.9)	15(1.4)	52(2.9)	11(7.2)	
Undetermined, n (%)	159(3.6)	39(2.7)	93(8.5)	24(1.3)	3(2.0)	
Tertiary hospital	9239 (92.6)	4180 (87.0)	2444 (95.8)	2024 (100.0)	591 (100.0)	< 0.001

^a^ percentage based on non-missing values

^b^ stroke etiology was available in 4474 patients, including 1438 patients in east area, 1099 patients in northeast area, 1785 patients in central area and 152 patients in west area. There were 5499 patients who had a missing value of stroke etiology in our analysis

GCS: Glasgow Coma Scale; NIHSS: National Institutes of Health Stroke Scale; IQR: interquartile ranges; TOAST: Trial of Org 10,172 in Acute Stroke Treatment

### Treatment pattern

As shown in Table 2, 429 (4.3%) patients received IVT. Of them, 363 (3.6%) received rt-PA, and 66 (0.7%) received urokinase. There were 9363 (93.9%) patients who received antiplatelet agents. Of them, 4997 cases (50.1%) received more than one kind of antiplatelet drug. During hospitalization, 1063 (10.7%) received anticoagulants, 6828 (68.5%) received statin, and 5112 (51.2%) received human urinary kallidinogenase (HUK).

**Table 2:** Pharmacotherapy of acute ischemic stroke in hospital by regions

	All (*N* = 9973)	East (*N* = 4806)	Northeast (*N* = 2552)	Central (*N* = 2024)	West (*N* = 591)	*P*
IVT	429 (4.3)	147 (3.1)	205 (8.0)	53 (2.6)	24 (4.1)	< 0.001
rt-PA	363 (3.6)	126 (2.6)	175 (6.9)	44 (2.2)	18 (3.0)	< 0.001
Urokinase	66 (0.7)	21 (0.4)	30 (1.2)	9 (0.4)	6 (1.0)	0.001
Antiplatelet agents	9363 (93.9)	4597 (95.7)	2324 (91.1)	1874 (92.6)	568 (96.1)	< 0.001
Aspirin	8186 (82.1)	3956 (82.3)	2024 (79.3)	1666 (82.3)	540 (91.4)	< 0.001
Clopidogrel	6106 (61.2)	3170 (66.0)	1345 (52.7)	1260 (62.2)	331 (56.0)	< 0.001
Cilostazol	83 (0.8)	63 (1.3)	16 (0.6)	3 (0.1)	1 (0.2)	< 0.001
Tirofiban	50 (0.5)	12 (0.2)	19 (0.7)	17 (0.8)	2 (0.3)	0.003
Ticagrelor	7 (0.07)	4 (0.08)	1 (0.04)	1 (0.05)	1 (0.2)	0.70
Anticoagulants	1063 (10.7)	480 (10.0)	221 (8.7)	311 (15.4)	51 (8.6)	< 0.001
Heparin	149 (1.5)	48 (1.0)	22 (0.9)	76 (3.8)	3 (0.5)	< 0.001
LMWH	713 (7.1)	333 (6.9)	151 (5.9)	207 (10.2)	22 (3.7)	< 0.001
Warfarin	184 (1.8)	102 (2.1)	11 (0.4)	40 (2.0)	31 (5.2)	< 0.001
NOAC	102 (1.0)	52 (1.1)	42 (1.6)	7 (0.3)	1 (0.2)	< 0.001
Fondaparinux sodium	2 (0.02)	0 (0.0)	1 (0.04)	1 (0.05)	0 (0.0)	0.33
Statin	6828 (68.5)	3502 (72.9)	1602 (62.8)	1372 (67.8)	352 (59.6)	< 0.001
Human urinary kallidinogenase	5112 (51.2)	2374 (49.4)	1761 (69.0)	908 (44.9)	68 (11.5)	< 0.001

Values are reported as n (%)

IVT, intravenous thrombolysis; rt-PA, recombinant tissue plasminogen activator; LMWH, low molecular weight heparin; NOAC, non-vitamin K oral anticoagulants

After adjusting for patient characteristics and hospital level, we still observed the disparity of the treatment patterns among regions. Compared with the eastern region, patients from the northeastern region were more likely to receive IVT (OR = 3.17, 95%CI: 2.53–3.99, *P* < 0.001) and HUK (OR = 2.30, 95%CI: 2.07–2.56, *P* < 0.001) and less likely to receive antiplatelets (OR = 0.46, 95%CI: 0.38–0.57, *P* < 0.001) and statins (OR = 0.60, 95%CI: 0.54–0.67, *P* < 0.001); the patients in the central region had lower uses of antiplatelets (OR = 0.55, 95%CI: 0.44–0.69, *P* < 0.001), statins (OR = 0.73, 95%CI: 0.65–0.82, *P* < 0.001), and HUK (OR = 0.76, 95%CI: 0.68–0.84, *P* < 0.001), but a higher use of anticoagulants (OR = 1.63, 95%CI: 1.38–1.92, *P* < 0.001); the western region had lower uses of statins (OR = 0.51, 95%CI: 0.43–0.61, *P* < 0.001) and HUK (OR = 0.12, 95%CI: 0.09–0.15, *P* < 0.001). The results of univariate and multivariate analysis are presented in Table 3.

**Table 3:** Association of pharmacotherapy for acute ischemic stroke and hospital regions

	Univariate analysis		Multivariate analysis	
	*P*	OR (95%CI)	*P*	OR (95%CI)
**IVT**				
East	Ref.		Ref.	
Northeast	< 0.001	2.77 (2.23–3.44)	< 0.001	3.17 (2.53–3.99)
Central	0.33	0.85 (0.62–1.17)	0.52	0.90 (0.65–1.25)
West	0.19	1.34 (0.86–2.08)	0.15	1.39 (0.89–2.18)
**Antiplatelet agents**				
East	Ref.		Ref.	
Northeast	< 0.001	0.46 (0.38–0.56)	< 0.001	0.46 (0.38–0.57)
Central	< 0.001	0.57 (0.46–0.71)	< 0.001	0.55 (0.44–0.69)
West	0.61	1.12 (0.72–1.74)	0.62	1.12 (0.72–1.75)
**Anticoagulants**				
East	Ref.		Ref.	
Northeast	0.07	0.85 (0.72–1.01)	0.004	0.77 (0.64–0.92)
Central	< 0.001	1.64 (1.40–1.91)	< 0.001	1.63 (1.38–1.92)
West	0.30	0.85 (0.63–1.15)	0.21	0.82 (0.60–1.12)
**Statin**				
East	Ref.		Ref.	
Northeast	< 0.001	0.63 (0.57–0.70)	< 0.001	0.60 (0.54–0.67)
Central	< 0.001	0.78 (0.70–0.88)	< 0.001	0.73 (0.65–0.82)
West	< 0.001	0.55 (0.46–0.65)	< 0.001	0.51 (0.43–0.61)
**Human urinary kallidinogenase**				
East	Ref.		Ref.	
Northeast	< 0.001	2.28 (2.06–2.52)	< 0.001	2.30 (2.07–2.56)
Central	0.001	0.83 (0.75–0.93)	< 0.001	0.76 (0.68–0.84)
West	< 0.001	0.13 (0.10–0.17)	< 0.001	0.12 (0.09–0.15)

IVT, intravenous thrombolysis

Adjusted for demographics (age, gender, medical insurance), medical history (hypertension, diabetes, dyslipidemia, atrial fibrillation, history of stroke including previous ischemic stroke and/or hemorrhagic stroke, coronary artery disease, and history of tumors), clinical features of the index stroke including National Institutes of Health Stroke Scale score and Glasgow Coma Scale score on admission and hospital level

There were 429 stroke patients with AF. Of them, 189 (44.1%) received anticoagulants during hospitalization. Among the 9544 patients without AF, 874 (9.2%) received anticoagulants. There were no differences among regions regarding the use of anticoagulants in patients with AF (*P* = 0.08). After adjusting the confounders, there were differences regarding the use of anticoagulants in patients without AF in the northeast (OR = 0.78, 95%CI: 0.64–0.95, *P* = 0.013) and central (OR = 1.62, 95%CI: 1.36–1.92, *P* < 0.001) regions compared with the eastern region (Table 4).

**Table 4:** Use of anticoagulants in acute ischemic stroke patients with and without atrial fibrillation by regions

	All	East	Northeast	Central	West	*P*
**With atrial fibrillation**	**n = 429**	**n = 204**	**n = 143**	**n = 61**	**n = 21**	
Anticoagulants	189 (44.1)	93 (45.6)	52 (36.4)	33 (54.1)	11 (52.4)	0.08
Heparin	5 (1.2)	1 (0.5)	2 (1.4)	2 (3.3)	0	0.264
LMWH	84 (19.6)	40 (19.6)	21 (14.7)	19 (31.1)	4 (19.0)	0.068
Warfarin	72 (16.8)	43 (21.1)	3 (2.1)	18 (29.5)	8 (38.1)	< 0.001
NOAC	57 (13.3)	27 (13.2)	28 (19.6)	2 (3.3)	0	0.001
Fondaparinux sodium	1 (0.2)	0	0	1 (1.6)	0	0.191
OR (95% CI)						
Crude		Ref.	0.68 (0.44–1.06)	1.41 (0.79–2.50)	1.31 (0.53–3.23)	
Adjusted		Ref.	0.65 (0.41–1.04)	1.42 (0.77–2.61)	1.21 (0.47–3.12)	
**Without atrial fibrillation**	**n = 9544**	**n = 4602**	**n = 2409**	**n = 1963**	**n = 570**	
Anticoagulants	874 (9.2)	387 (8.4)	169 (7.0)	278 (14.2)	40 (7.0)	< 0.001
Heparin	144 (1.5)	47 (1.0)	20 (0.8)	74 (3.8)	3 (0.5)	< 0.001
LMWH	629 (6.6)	293 (6.4)	130 (5.4)	188 (9.6)	18 (3.2)	< 0.001
Warfarin	112 (1.2)	59 (1.3)	8 (0.3)	22 (1.1)	23 (4.0)	< 0.001
NOAC	45 (0.5)	25 (0.5)	14 (0.6)	5 (0.3)	1 (0.2)	0.280
Fondaparinux sodium	1 (0.01)	0	1 (0.04)	0	0	0.518
OR (95% CI)						
Crude		Ref.	0.82 (0.68–0.99)*	1.80(1.53–2.12)**	0.82 (0.59–1.15)	
Adjusted		Ref.	0.78 (0.64–0.95)*	1.62(1.36–1.92)**	0.76 (0.54–1.06)	

*P < 0.05, **P < 0.01

LMWH: low molecular weight heparin; NOAC: non-vitamin K oral anticoagulants

Adjusted for demographics (age, sex, medical insurance), medical history (hypertension, diabetes, dyslipidemia, history of stroke including previous ischemic stroke and/or hemorrhagic stroke, coronary artery disease, and history of tumors), clinical features of the index stroke including National Institutes of Health Stroke Scale score and Glasgow Coma Scale score on admission, and hospital level

### Functional outcome

The mRS score was available for 9345 (93.7%) patients at 3 months and 8673 (87.0%) at 12 months. The proportions of poor outcomes were 20.7% at 3 months and 15.8% at 12 months.

After adjusting for the confounders, patients in the northeastern and central region had significantly lower risk of poor functional outcome at 3 months (OR = 0.85, 95%CI: 0.74–0.98, *P* = 0.026; OR = 0.69, 95%CI: 0.60–0.81, *P* < 0.001) and at 12 months (OR = 0.84, 95%CI: 0.72–0.99, *P* = 0.035; OR = 0.81, 95%CI: 0.69–0.96, *P* = 0.013), comparing with those in the eastern region (Table 5).

**Table 5:** Poor outcomes of acute ischemic stroke patients and hospital regions

Poor outcome	All	East	Northeast	Central	West
At 3 months ^a^	1935 (20.7)	973 (22.3)	461 (19.2)	368 (18.5)	133 (22.9)
Crude		Ref.	0.83 (0.73–0.94)**	0.79 (0.69–0.91)**	1.03 (0.84–1.27)
Adjusted		Ref.	0.85 (0.74–0.98)*	0.69 (0.60–0.81)**	1.01 (0.80–1.27)
At 12 months ^a^	1373 (15.8)	682 (16.9)	315 (14.3)	290 (15.3)	86 (15.9)
Crude		Ref.	0.82 (0.71–0.94)**	0.89 (0.77–1.03)	0.93 (0.73–1.19)
Adjusted		Ref.	0.84 (0.72–0.99)*	0.81 (0.69–0.96)*	0.89 (0.68–1.16)

*P < 0.05, **P < 0.01, ^a^percentage based on non-missing values

Results are presented as n (%) and odds ratio (95% confidence interval)

Adjusted for demographics (age, sex, medical insurance), medical history (hypertension, diabetes, dyslipidemia, atrial fibrillation, history of stroke including previous ischemic stroke and/or hemorrhagic stroke, coronary artery disease, and history of tumors), clinical features of the index stroke including National Institutes of Health Stroke Scale score and Glasgow Coma Scale score on admission, and hospital level

## Discussion

This study investigated the status of pharmacotherapy for AIS and outcomes in China and explored the geographic variation in stroke care. In this study based on CASTOR database, the proportions of the use of IVT, antiplatelet agents, statin, anticoagulants, and human urinary kallidinogenase were 4.3%, 93.9%, 68.5%, 10.7%, and 51.2%, respectively. Poor functional outcome was observed in 20.7% of the patients at 3 month and 15.8% at 12 month. There were significant geographic variations in pharmacotherapy and outcome for AIS in China.

Since the NINDS trial in 1996 [19], IVT with rt-PA for AIS has been recommended by stroke guidelines and applied worldwide [10, 20–22]. The IVT rate is considered an important indicator of the quality of stroke care [5, 7, 8]. The present study showed that 4.3% of the patients with AIS received IVT in 2015–2017 in a multicenter registry covering China. Another study based on the Bigdata Observatory platform for Stroke of China (BOSC) reported an IVT rate of 5.64% between 2019 and 2020 [5]. Compared to the IVT rates of 2% in 2006, 2.4% in 2007–2008, and 2.5% in 2012–2013 [4, 6–8], there was a substantial improvement in the IVT rate for AIS in China in 2015–2017. A recent study found that the increased IVT rate with rt-PA was positively correlated with the number and density of stroke centers, suggesting that the stroke center certification launched by the China Stroke Prevention Project Committee (CSPPC) could promote the use of IVT [23]. Notably, there was still a gap compared with the developed countries. For example, the IVT rate was 10.9% in the USA in 2018 [24]. Besides, a significant regional difference was another problem. Indeed, the IVT rates were 3.1%, 8.0%, 2.6%, and 4.1% in the eastern, northeastern, central, and western regions. The exact reasons for these differences need further investigation. Indeed, the economic status does not appear to be involved since the poorest region (western region) had an IVT rate of 4.1%. Similar results were observed by Chen et al. [25]. The knowledge, attitude, and practice (KAP) of IVT could be involved and should be investigated. Nevertheless, these data indicate that more efforts are still needed to improve the IVT use for AIS in China.

Besides rt-PA, urokinase is another choice for IVT recommended by the Chinese stroke guideline [10]. A study reported the efficacy and safety of urokinase for AIS within 6 h of onset in China [26]. Compared with rt-PA, urokinase is much less expensive and has a longer time window. Although this study showed that rt-PA was used in a higher proportion of AIS patients receiving IVT, urokinase had an important potential for IVT in rural areas. Nevertheless, besides being recommended by the Chinese guidelines [10], the evidence for using urokinase in AIS remains relatively low, limiting its use.

Antiplatelet therapy plays an important role in treating AIS and is recommended by many stroke guidelines [10, 20–22]. The use of aspirin within 48 h after stroke onset can significantly reduce the risk of stroke recurrence and mortality [27, 28]. In the present study, 93.9% of the patients in the cohort had received antiplatelet agents, significantly higher than the proportion of 81% in 2006 reported by another registry study in China [29] and similar with a rate of 96.3% in USA [30]. In addition, the present study showed the use of antiplatelet therapy in different regions was close, ranging from 91.1 to 96.1%. Although statistically significant differences were observed among regions, the differences were small and possibly due to the large sample size. Further analysis showed aspirin and clopidogrel were the most commonly used antiplatelet agents. And about 50% of the patients had received more than one kind of antiplatelet drug during hospitalization, which could be due to 45% patients in our database had a NIHSS score ≤ 3 at baseline. The current guidelines recommend dual antiplatelet therapy in minor stroke/transient ischemic attack (TIA) and stroke patients with severe intracranial artery stenosis based on randomized clinical trials [10, 20–22]. The widespread use in clinical practice in China warrants further evaluation of the effectiveness and safety of dual antiplatelet therapy in the real world.

A growing number of studies showed that statins could improve the outcomes of patients with AIS [31, 32]. Indeed, besides its lipid-lowering effects, statins possess pleiotropic effects on inflammation, oxidative stress, and platelet activation. In the present study, up to 68.5% of the patients with AIS received statin, much higher than 31% observed in 2006 [29]. Even the lowest proportion reported in the western region was nearly 60%. Still, statin use was lower in all regions when compared with the eastern region, probably because the eastern region has the highest economic status. Nevertheless, the results indicated a significant improvement in the lipid-lowing treatment of AIS patients in China.

The guidelines do not recommend anticoagulation for general patients with AIS due to a lack of conclusive evidence, but recommend anticoagulation for those patients with both AIS and AF [10, 20–22, 33–35]. Nevertheless, anticoagulation therapy is still widely used in clinical practice. An international, multicenter study showed that 8% of AIS patients were fully anticoagulated in the first week after stroke [36], consistent with the results of registry studies in the USA and Australia [37, 38]. In the present study, 10.7% of the patients received anticoagulation therapy, similar to the literature [37, 38]. In 2006, a prospective, multicenter, hospital-based registry showed that 18.8% of the patients with AIS received anticoagulation agents in China [39], while the proportion of anticoagulation therapy was as high as 33.7% in 2013 [40]. Therefore, the present study observed a significantly lower use of anticoagulation therapy, suggesting better compliance with the guidelines. Still, compared with the eastern region, the central region had significantly higher use of anticoagulants. The reasons for this higher use should be investigated. Besides, in this present study, only 44.1% of the patients with AIS and AF received anticoagulation agents, which was much lower than that in USA [4, 9]. The lower rate of AF than that in developed countries [4] could be due to under diagnosis. Therefore, the screening and treatment of cardiogenic factors in managing AIS should be strengthened in China.

As for the options of anticoagulants, LMWH was still the main anticoagulant in the acute phase of ischemic stroke, followed by oral anticoagulants. In terms of oral anticoagulants, although NOACs have recently replaced warfarin as the more commonly used anticoagulant in European and North American countries (58-66.5%) [41], warfarin was more frequently prescribed than NOAC (64.3% vs. 35.7%) in the present study. The regional differences in the use of NOACs in China were apparent. The proportion in the northeastern and eastern regions was higher than in the central and western regions. We speculated the economic factors might contribute to the disparity.

HUK can enhance collateral circulation and angiogenesis and improve cerebral perfusion and functional outcomes after AIS [42–44]. HUK has been approved for managing AIS by China’s State Food and Drug Administration [10]. The present study showed that HUK was used widely in China, and 51.2% of patients received this drug in our cohort. HUK was more frequently used in the northeastern region compared with the eastern region, which could be due to the local medication experience. On the other hand, it was less used in the central and western regions, probably due to economic factors.

In this study, the proportions of patients with a poor outcome at 3 and 12 month were 20.7% and 15.8%, respectively. Compared with other studies [45], the relatively lower risk of poor outcome in our study could attribute to a high proportion of patients with mild symptom. There were also geographic variations among regions. The lower risk of poor functional outcomes were observed in patients from the northeastern and central regions, while the risk was similar between eastern and western region. This was consistent with the higher medical insurance coverage rate in the northeastern and central regions in our cohort, suggesting the importance of medical insurance in the management of AIS.

This study had several limitations. First, the enrollment of the hospitals was not randomized, and there is a possibility of selection bias. Nevertheless, given the broad and extensive geographic coverage of the study, this study provided some useful information on the current status of pharmacotherapy and prognosis of AIS in China. Second, the patients with AIS were admitted within 1 week of onset. Hence, the patients who died before admission or were not hospitalized were not included. In addition, 92.5% of the patients were from tertiary hospitals, which may limit the generalizability of the results. Third, this study focused on the patients admitted between 2015 and 2017. Since then, there has been a series of important progress in the management of AIS, especially in endovascular therapy. Further study is needed to follow up on the changes in the pharmacotherapy regimens for AIS in China. Fourth, we speculated the regional variations in the pharmacotherapy and prognosis of AIS might attribute to different medical insurance coverage, economic conditions, and guideline compliance based on previous literatures [46–48]. Additionally, the geographic location, weather and diet habit might also play a role on them. However, our study could not provide with direct evidences to support these hypotheses. The exact reasons for the regional variations need further investigation. Fifth, in our analysis, stroke etiology according to TOAST criteria was available in only 4474 patients. Therefore, we did not adjust for stroke etiology in multivariable analyses and we included stroke patients with AF as the appropriate candidates for anticoagulation treatment. However, it would be more reasonable to investigate antiplatelet and anticoagulation treatment by different TOAST classification.

## Conclusion

Based on a large, multicenter registry study of acute ischemic stroke in China (the CASTOR database), this study described the current status of pharmacotherapy and the outcomes of AIS in China. There was a relatively high proportion of antiplatelet agent and statin use. The IVT rate also appeared to have improved but was still lower than in developed countries. For patients with AIS and AF, there was insufficient use of anticoagulant agents. There was substantial regional variation in pharmacotherapy and prognosis of AIS in China.

## Data Availability

The data supporting the findings of this study are available within the article.

## Abbreviations

AF: Atrial fibrillation
AIS: Acute ischemic stroke
BOSC: Bigdata Observatory platform for Stroke of China
CSPPC: China Stroke Prevention Project Committee
GCS: Glasgow Coma Scale
HUK: Human urinary kallidinogenase
IVT: Intravenous thrombolysis
IQR: Interquartile ranges
LMWH: Low molecular weight heparin
mRS: Modified Rankin Scale
NIHSS: National Institutes of Health Stroke Scale
rt-PA: Recombinant tissue plasminogen activator
TIA: Transient ischemic attack
